# Chronic dexamethasone exposure retards growth without altering the digestive tract microbiota composition in goats

**DOI:** 10.1186/s12866-018-1253-1

**Published:** 2018-09-10

**Authors:** Canfeng Hua, Yali Geng, Qu Chen, Liqiong Niu, Liuping Cai, Shiyu Tao, Yingdong Ni, Ruqian Zhao

**Affiliations:** Key Laboratory of Animal Physiology & Biochemistry, Nanjing Agricultural University, Nanjing, 210095 People’s Republic of China

**Keywords:** Chronic stress, Dexamethasone, Growth, Micobiota composition, Goat

## Abstract

**Background:**

Dexamethasone (Dex), an artificially synthetic cortisol substitute, is commonly used as an anti-inflammatory drug, and is also employed to mimic the stress state experimentally. It is well known that chronic stress disturbs the gut microbiota community and digestive functions. However, no relevant studies have been conducted in ruminants.

**Results:**

In this study, a low dosage of Dex (0.2 mg/kg body weight, Dex group, *n* = 5) was consecutively injected intramuscularly for 21 days to simulate chronic stress in growing goats. Goats were injected with saline (0.2 mg/kg body weight) as the control group (Con, n = 5). Dex-treated goats showed a higher number of white blood cells and blood glucose levels (*p* < 0.01), but lower dry matter intake (DMI) and body weight (*p* < 0.01) than those of saline-injected goats. Plasma cortisol concentration decreased significantly in response to the Dex injection compared to the control (*p* < 0.05). The Dex treatment did not change most ruminal volatile fatty acid (VFAs) concentrations before the morning feeding after 1–21 days of treatment (*p* > 0.05); however, ruminal VFA concentrations decreased dramatically 2, 4, 6, and 8 h after the morning feeding on day 21 of the Dex injections. In this study, chronic Dex exposure did not alter the community structure of microbes or methanogenes in the rumen, caecum, or colonic digesta. Only *Prevotella* increased on days 7 and 14 of Dex treatment, but decreased on day 21, and *Methanosphaera* was the only genus of methanogene that decreased.

**Conclusions:**

Our results suggest that chronic Dex exposure retards growth by decreasing DMI, which may be mediated by higher levels of blood glucose and lower ruminal VFA production. Microbiota in the digestive tract was highly resistant to chronic Dex exposure.

**Electronic supplementary material:**

The online version of this article (10.1186/s12866-018-1253-1) contains supplementary material, which is available to authorized users.

## Background

Cortisol is a stress hormone synthesized and released by the pituitary gland under stress conditions [1]. Stress induces metabolic disorders, such as diabetes and cardiovascular disease [2, 3]. Acute stress-induced hyperglycemia is observed in many situations, such as sepsis, myocardial infarct, shock, stroke, and trauma [4–7]. A major mechanism by which cortisol regulates glucose metabolism is to inhibit insulin signaling, which promotes glucose transporter 4 expression on the cell surface [8, 9]. Stress has significant negative impacts on feeding and ultimately livestock productivity, which reduce efficiency and energy for physical maintenance [10]. Stress inhibits growth hormone secretion by decreasing circulating somatostatin [2]. Dexamethasone (Dex) has been used to simulate stress conditions in non-ruminant and ruminant animals [11].

Microbiota play an important role in maintaining gut health and body homeostasis. Microbiota affect mucosal defense and inhibit the ability of enteric pathogens to colonize. Bäckhed et al. and Ley et al. reported that intestinal microbiota are associated with obesity and diabetes in germ-free mice [12, 13]. Many metabolites in the intestine are synthesized by microbes, such as vitamins K and B complex [14].

Prolonged stressors can radically change the composition of the intestinal microbiota and significantly increase circulating levels of interleukin-6 and monocyte chemoattractant protein-1, which are correlated with changes in *Coprococcus*, *Pseudobutyrivibrio*, and *Dorea* populations [15]. This disruption of microbiota increases tumor necrosis factor-alpha gene expression in colonic tissue and elevates susceptibility to enteric pathogens [16]. Söderholm et al. [17] suggested that stress can induce intestinal inflammation by impairing mucosal defenses against luminal bacteria. Stress affects the integrity of the mucosal immune system by increasing CD4+ T and CD19+ B cells in the large intestine [18]. Pitlik and Koren [19] hypothesized that all diseases alter microbiome composition, turning a healthy microbiome into a disease pathobiome.

The methane concentration in the atmosphere has increased since the agricultural and industrial revolution over 200 years ago. Methane emissions from ruminant livestock (cattle, sheep, and goats), which is produced by methanogenic archaea, is a major contributor to anthropogenic greenhouse gas emissions worldwide [20]. Ruminants emit about 100 million tons of methane per year, which corresponds to ~ 20% of global methane emissions. Methane is also a net loss of feed energy to the animal [21]. However, research on how stress impacts methanogenes is lacking.

Many studies have investigated how the host impacts the intestinal microbiome [19, 22]. However, studies about stress impacting microbiota in goats are lacking. Therefore, the object of present study is to investigate the influence of chronic stress on the microbiota in the rumen, caecum, and colon of goats.

## Results

### Changes in the number of blood cells in response to Dex

The number of white blood cells (WBCs) (*p* < 0.01), neutrophils, lymphocytes, and eosinophils (*p* < 0.05) increased significantly after 14 days (*p* < 0.05) and 21 days of Dex injections (Table 1). The number of red blood cells increased markedly after 14 days of Dex treatment (*p* < 0.01), and showed a trend to increase on day 21 of Dex treatment compared to that in control goats (0.05 < *p* < 0.1) (Table 1). Moreover, mean corpuscular hemoglobin concentration decreased after 14 and 21 days of Dex treatment (0.05 < *p* < 0.1) (Table 1).

**Table 1:** The component of blood cell

	Day 14		Day 21	
	Con	Dex	Con	Dex
WBC(10^9/L)	13.34±1.19	21.66±2.12^*^	10.13±1.64	16.86±0.50^**^
Neu#(10^9/L)	7.99±1.03	13.83±1.93^*^	5.41±1.07	9.88±0.36^**^
Lym#(10^9/L)	4.64±0.32	6.90±0.50^**^	4.17±0.63	6.07±0.19^*^
Mon#(10^9/L)	0.45±0.08	0.60±0.02	0.40±0.01	0.48±0.03^#^
Eos#(10^9/L)	0.14±0.01	0.22±0.02^*^	0.09±0.02	0.18±0.03^*^
Bas#(10^9/L)	0.12±0.02	0.16±0.02	0.09±0.02	0.11±0.01
RBC(10^12/L)	14.88±0.77	18.60±0.55^**^	15.45±0.75	17.17±0.33^#^
HGB(g/L)	95.25±6.12	99.80±3.53	86.25±4.03	87.20±1.36
HCT%	27.78±1.94	28.26±1.27	27.07±1.09	27.90±0.75
MCV(fL)	16.33±0.61	15.22±0.45	18.92±1.13	15.83±0.34^#^
MCH(pg)	5.98±0.27	5.36±0.11^#^	5.40±0.08	5.08±0.12^#^
MCHC(g/L)	344.75±6.22	353.60±5.05	297.40±7.27	312.40±5.23
RDW-CV%	23.85±0.77	25.03±0.83	24.38±0.99	24.30±0.57
RDW-SD(fL)	15.23±0.80	15.90±0.47	18.22±1.39	16.57±0.03

*WBC* White blood cells, *Neu* Neutrophil, *Lym* Lymphocyte, *Mon* Monocyte, *Eos* Eosinophilic, *Bas* Basophil, *RBC* Red blood cells, *HGB* Hemoglobin, *HCT* Hematocrit, *MCV* Mean corpuscular volume, *MCH* Mean corpuscular hemoglobin, *MCHC* Mean corpuscular hemoglobin concentration, *RDW-CV* Red blood cell distribution width, *RDW-SD* Standard deviation of RBC distribution width. Means ± SE are plotted; #*p*<0.10, *p<0.05, **p<0.01 versus Con group

### Changes in dry matter intake, body weight, and blood glucose concentration in response to Dex

**Fig. 1:**
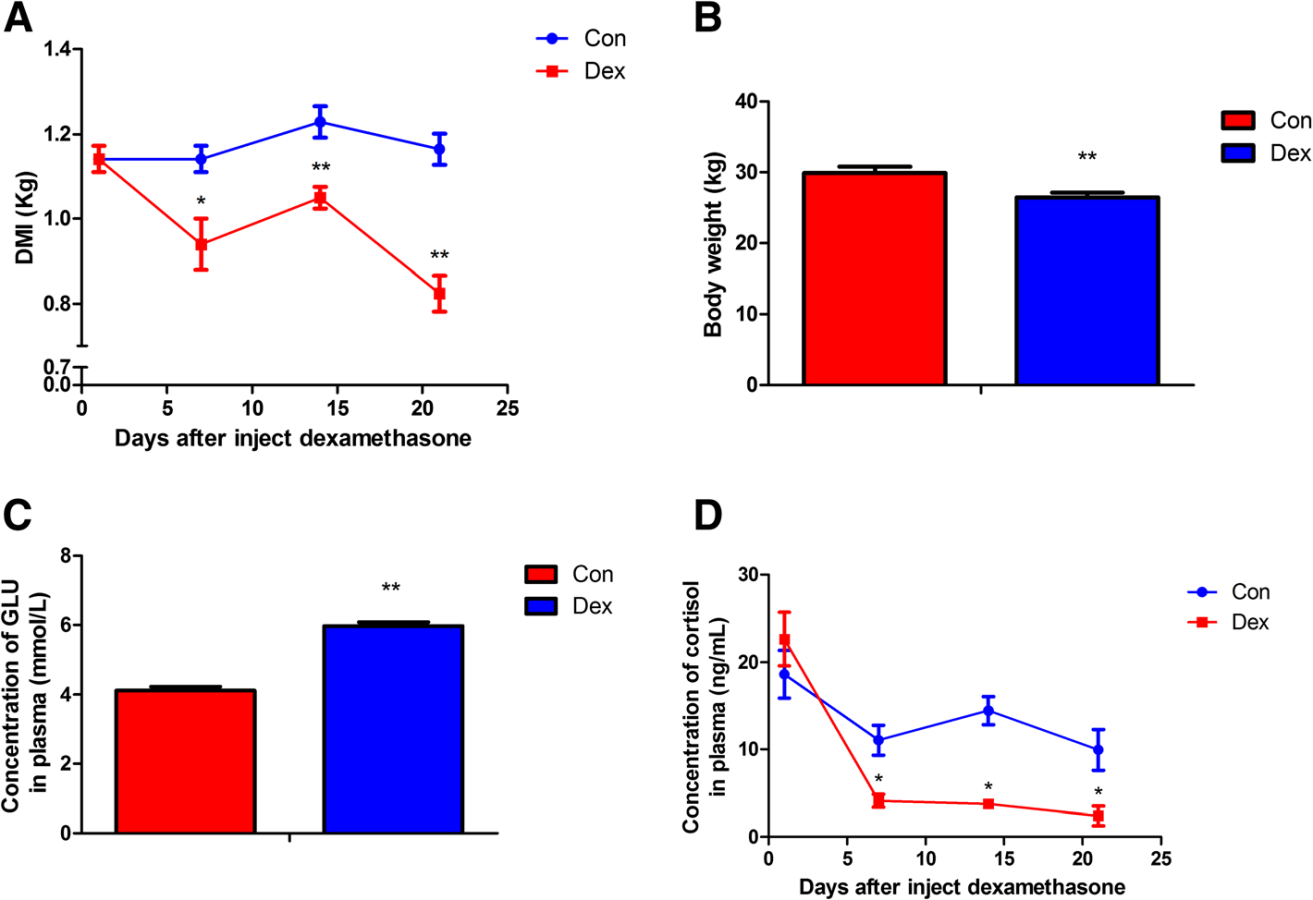
**a** Dynamic alteration of DMI from goat treated with Dex or Con for 21 days. **b** Body mass of goats at the 21th day of the experiment. **c** Concentration of glucose in plasma at the 21th day of the experiment. **d** Dynamic changes in the concentration of plasma cortisol from goat treat with Dex or Con for 21 days. Means ± SE are plotted; ^#^*p* < 0.10, *p < 0.05, **p < 0.01 versus Con group

As shown in Fig. 1a, dry matter intake (DMI) (*p* < 0.05) and body weight (*p* < 0.01) decreased significantly after the Dex injections compared to those in the control group (Fig. 1a). Blood glucose level increased significantly after 21 days of Dex injections (*p* < 0.05) (Fig. 1c). However, blood cortisol level decreased significantly in response to Dex treatment (*p* < 0.05) (Fig. 1d).

### Ruminal pH and urea, ammonia, and VFA concentrations

**Fig. 2:**
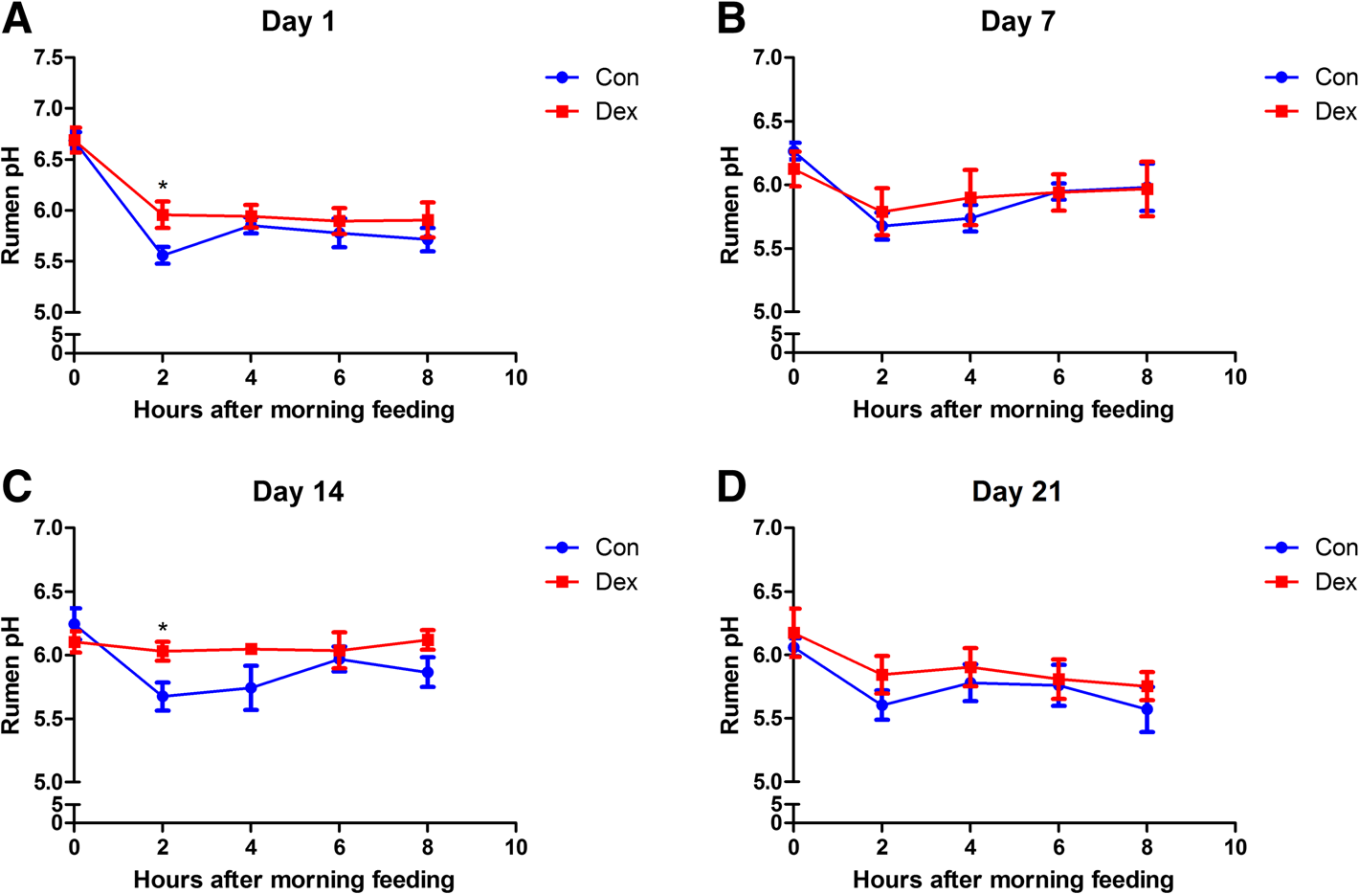
Dynamic alteration of pH in rumen fluid from goat treated with Dex or Con, at 1, 7, 14, 21 day, after morning feeding. **a** Day 1. **b** Day 7. **c** Day 14. **d** Day 21. Means ± SE are plotted; ^#^*p* < 0.10, *p < 0.05, **p < 0.01 versus Con group

**Fig. 3:**
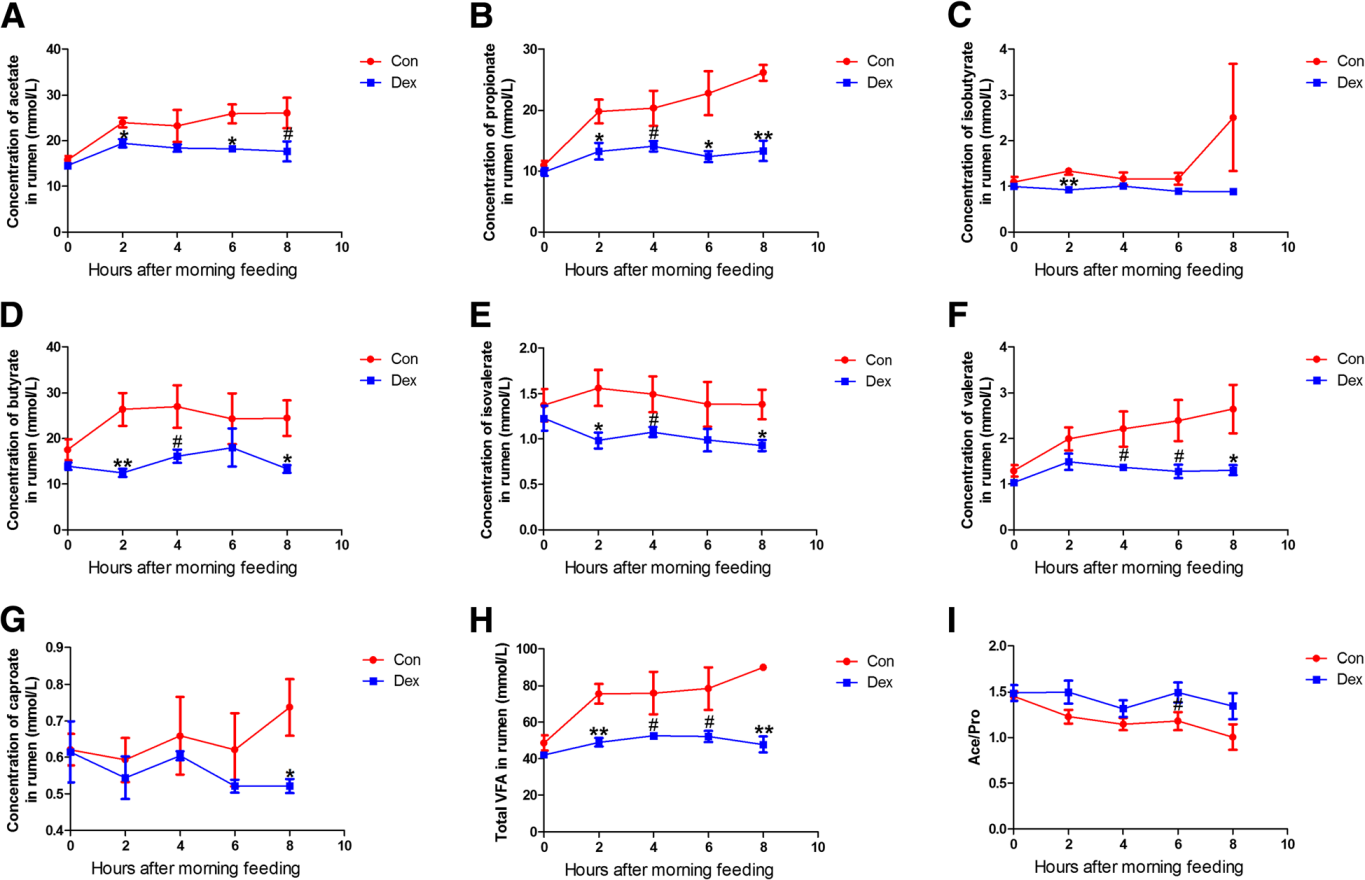
Dynamic alteration of VFAs in rumen from goat treated with Dex or Con, at the 21th day, after morning feeding. **a** Acetate. **b** Propionate. **c** isobutyrate. **d** Butyrate. **e** Isovalerate. **f** Valerate. **g** Caproate. **h** Total VFAs. **i** Acetate/ Propionate. Means ± SE are plotted; ^#^*p* < 0.10, *p < 0.05, **p < 0.01 versus Con group

Dynamic changes in ruminal pH were detected at 0, 2, 4, and 6 h after the morning feeding on days 1, 7, 14, and 21 of the Dex injections. These results show that Dex increased ruminal pH. Ruminal pH increased significantly 2 h after the morning feeding on days 1 and 14 of Dex treatment compared to that in the control (*p* < 0.05) (Fig. 2a and c). The concentrations of ruminal urea and ammonia were not altered by Dex treatment compared to the control (*p* > 0.05) (Additional file 1: Figure S1 and Additional file 2: Figure S2). The VFA concentrations in ruminal fluid remained unchanged before the morning feeding on days 1, 7, 14, and 21 of the Dex injections (*p* > 0.05) (Additional file 3: Figure S3). However, the concentrations of total ruminal VFAs, acetate, propionate, isobutyrate, butyrate, isovalerate, valerate and caproate decreased significantly 0, 2, 4, 6, and 8 h after the morning feeding on day 21 of the Dex injections (*p* < 0.05) (Fig. 3a–h). In contrast, the acetate/propionate ratio was unchanged by Dex treatment (*p* > 0.05) (Fig. 3i).

### Changes in VFA concentrations in feces, caecum, and the colonic digesta

The concentrations of fecal butyrate and total VFAs decreased significantly in the Dex group compared to those in the control (*p* < 0.05) (Additional file 4: Figure S4), whereas the acetate/propionate ratio remained unchanged by Dex (*p* > 0.05) (Fig. 3i). Only isovalerate concentration in the colonic digesta increased significantly in response to Dex (*p* < 0.05), whereas the other VFAs and total VFA contents in the colonic and caecal digesta were not affected by the 21-day Dex treatment (*p* > 0.05) (Table 2). None of the fecal VFAs changed, but butyrate decreased significantly after 14 days of Dex administration, leading to a reduction in total VFAs (*p* < 0.05) (Additional file 4: Figure S4D and H).

**Table 2:** The component of VFA in caecum and colon

	Caecum		Colon	
Parameters	Con	Dex	Con	Dex
Acetate(mmol/L)	10.86±1.15	10.13±0.92	10.04±0.67	9.91±0.53
Propionate(mmol/L)	8.31±1.21	6.39±0.72	6.01±0.82	5.72±0.62
Isobutyrate(mmol/L)	0.72±0.12	0.62±0.09	0.51±0.04	0.67±0.18
Butyrate(mmol/L)	6.03±1.45	4.24±0.48	3.84±0.75	3.09±0.26
Isovalerate(mmol/L)	0.51±0.09	0.56±0.07	0.38±0.04	0.53±0.03^*^
Valerate(mmol/L)	1.21±0.28	1.02±0.15	0.71±0.13	0.72±0.10
Caproate(mmol/L)	1.01±0.40	0.52±0.13	0.47±0.02	1.56±0.54
Total(mmol/L)	28.65±3.93	23.69±2.81	25.64±0.41	22.19±1.38
Ace/Pro	1.33±0.08	1.61±0.09^#^	1.75±0.14	1.78±0.10

*Ace/Pro* Acetate/propionate. Means ± SE are plotted; #*p*<0.10, *p<0.05, **p<0.01 versus Con group

### The composition of microbiota in ruminal fluid, caeca, and colonic digesta

**Fig. 4:**
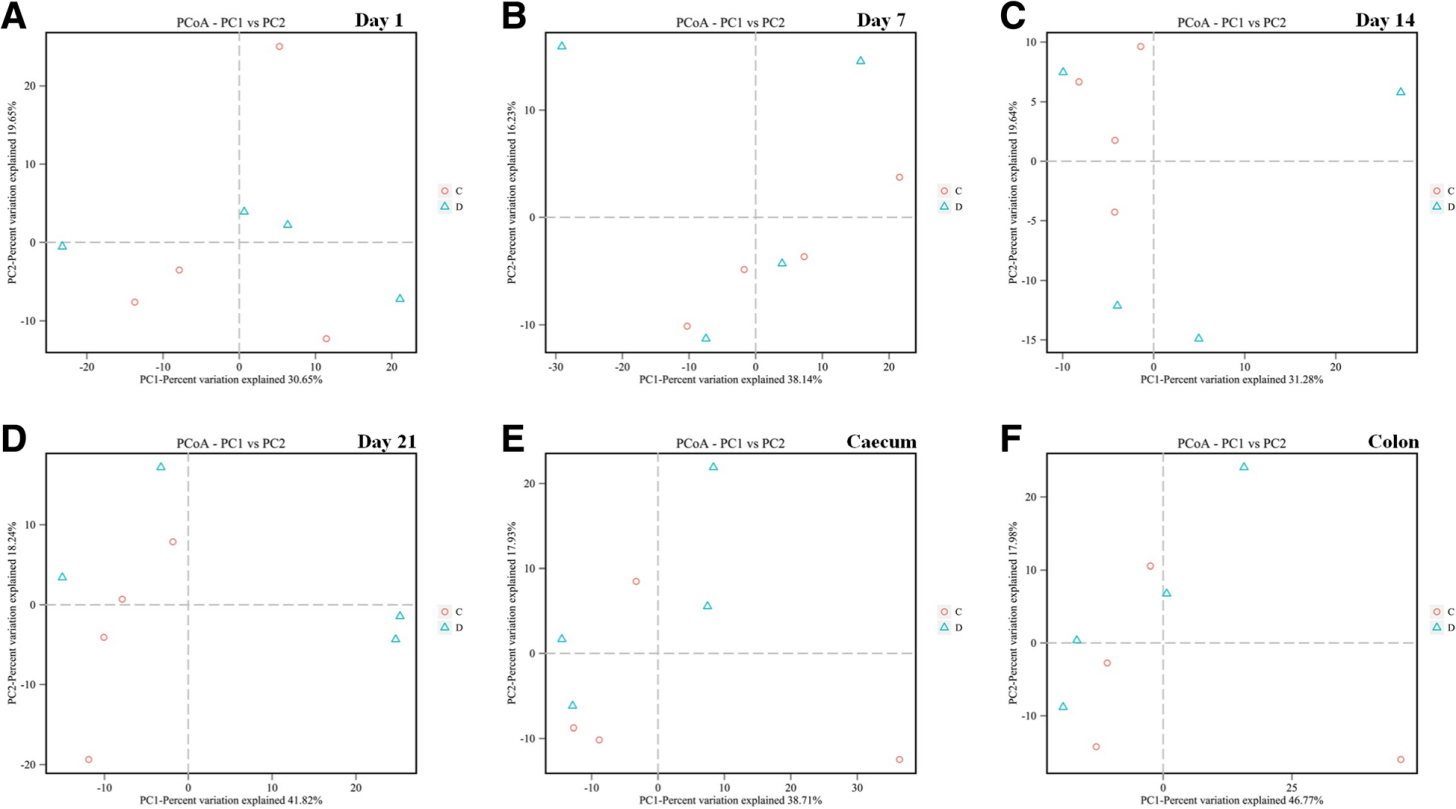
Unweighted PCoA of bacteria microbiota in rumen fluid and the content of caecum and colon from goat treated with Dex or Con. **a** Day 1 in rumen. **b** Day 7 in rumen. **c** Day 14 in rumen. **d** Day 21 in rumen. **e** Content of caecum. **f** Content of colon

**Fig. 5:**
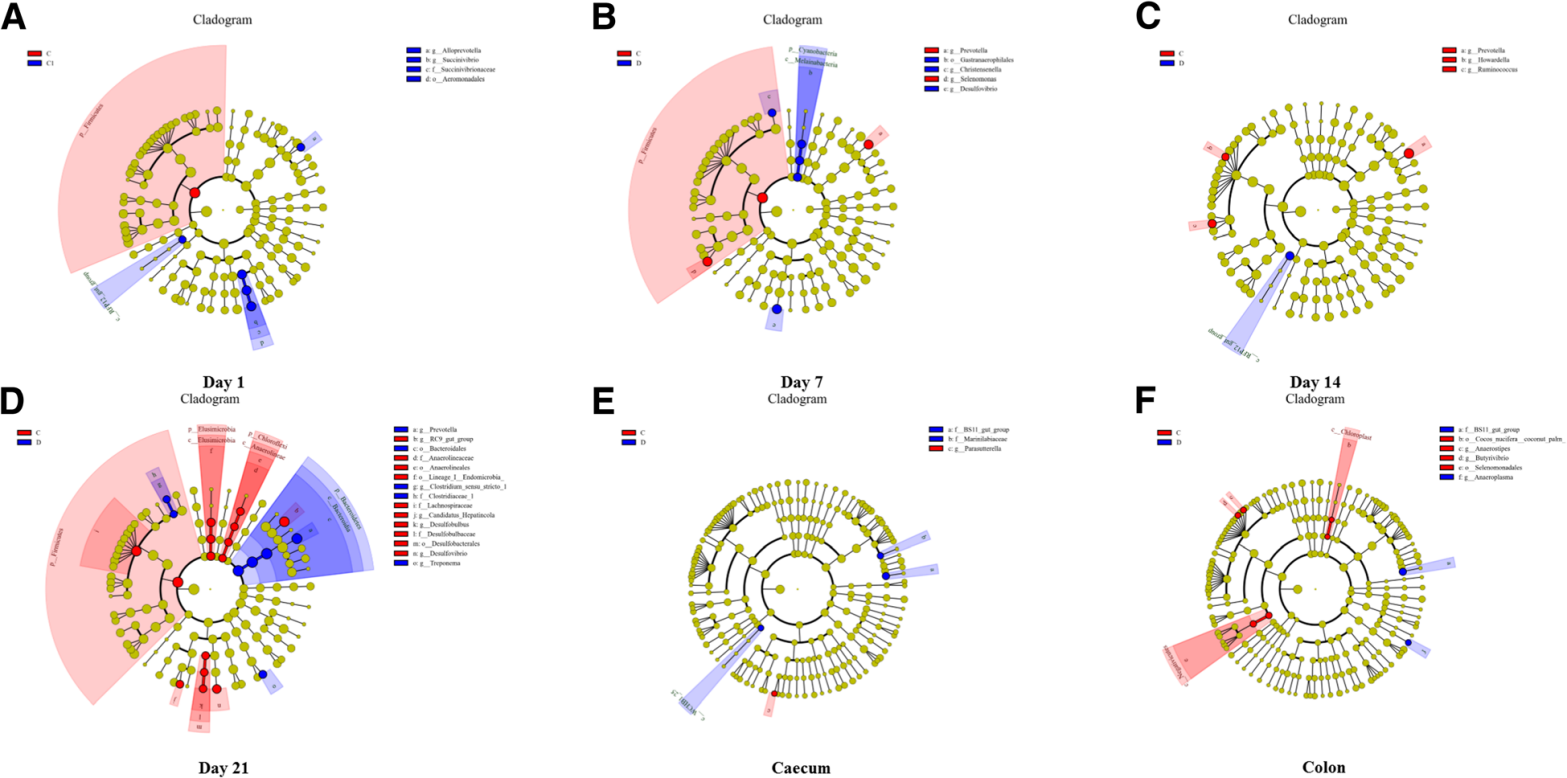
Cladograms, generated from LEfSe analysis, represent taxa enriched in Con (blue) or Dex (red) microbiota. The central point represents the root of the tree (Bacteria), and each ring represents the next lower taxonomic level (phylum through genus). The diameter of each circle represents the relative abundance of the taxon. When full identification was not possible, g_ or s_ alone was used for genus or species, respectively. **a** Day 1 in rumen. **b** Day 7 in rumen. **c** Day 14 in rumen. **d** Day 21 in rumen. **e** Content of caecum. **f** Content of colon

The composition of microbiota was analyzed by next-generation sequencing. Figure 4 shows the PCoA analysis of the bacteria, and the Con and Dex groups were not separated. The composition of microbiota was not affected by the Dex treatment, as the phylum composition did not change (Additional file 5: Figure S5A–F). *Prevotella*, and *Selenomonas* increased in abundance, whereas *Desulfovibrio* and *Christensenella* decreased in ruminal fluid after 7 days of Dex treatment (Fig. 5b). The abundances of *Prevotella*, *Howardella*, and *Ruminococcus* increased significantly after 14 days of Dex injections (Fig. 5c). The abundances of *Prevotella*, *Clostridium_sensu_stricto_1*, and *Treponema* were enhanced after 21 days of Dex treatment; however, the levels of *RC9_gut_group*, *Candidatus_Hepatincola*, *Desulfobulbus*, and *Desulfovibrio* decreased (Fig. 5d). *Parasutterella* in the caecal digesta decreased in response to Dex (Fig. 5e). The abundances of *Butyrivibrio* and *Anaerostipes* increased in colonic digesta, whereas that of *Anaeroplasma* decreased in response to Dex (Fig. 5f). At the family level, the abundance of the BS11_gut_group increased in both the caecum and colonic digesta after Dex administration (Fig. 5e and F).

### Changes in methanogens

**Fig. 6:**
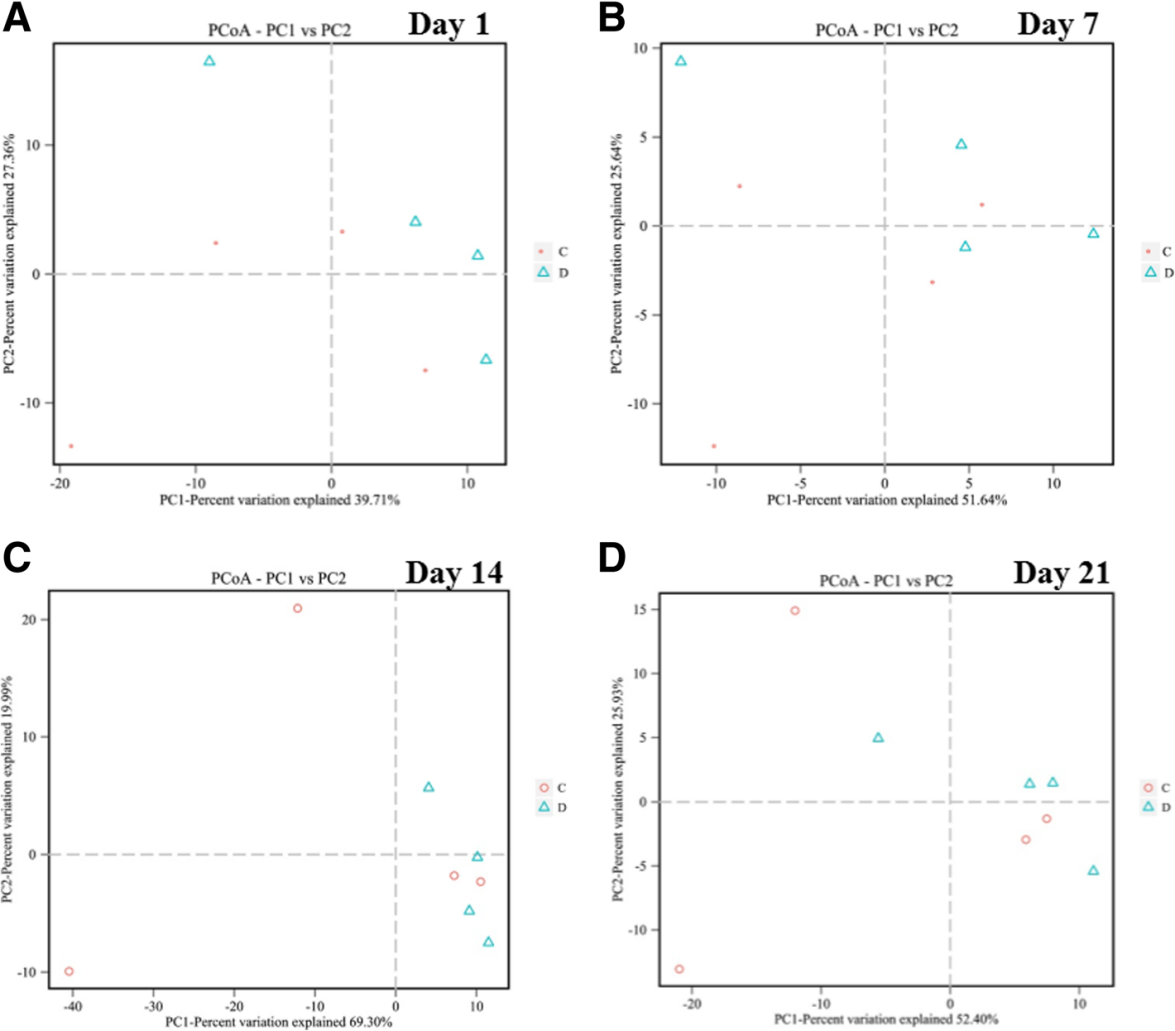
Unweighted PCoA of methanogens in rumen fluid and the content of caecum and colon from goat treated with Dex or Con. **a** Day 1 in rumen. **b** Day 7 in rumen. **c** Day 14 in rumen. **d** Day 21 in rumen

**Fig. 7:**
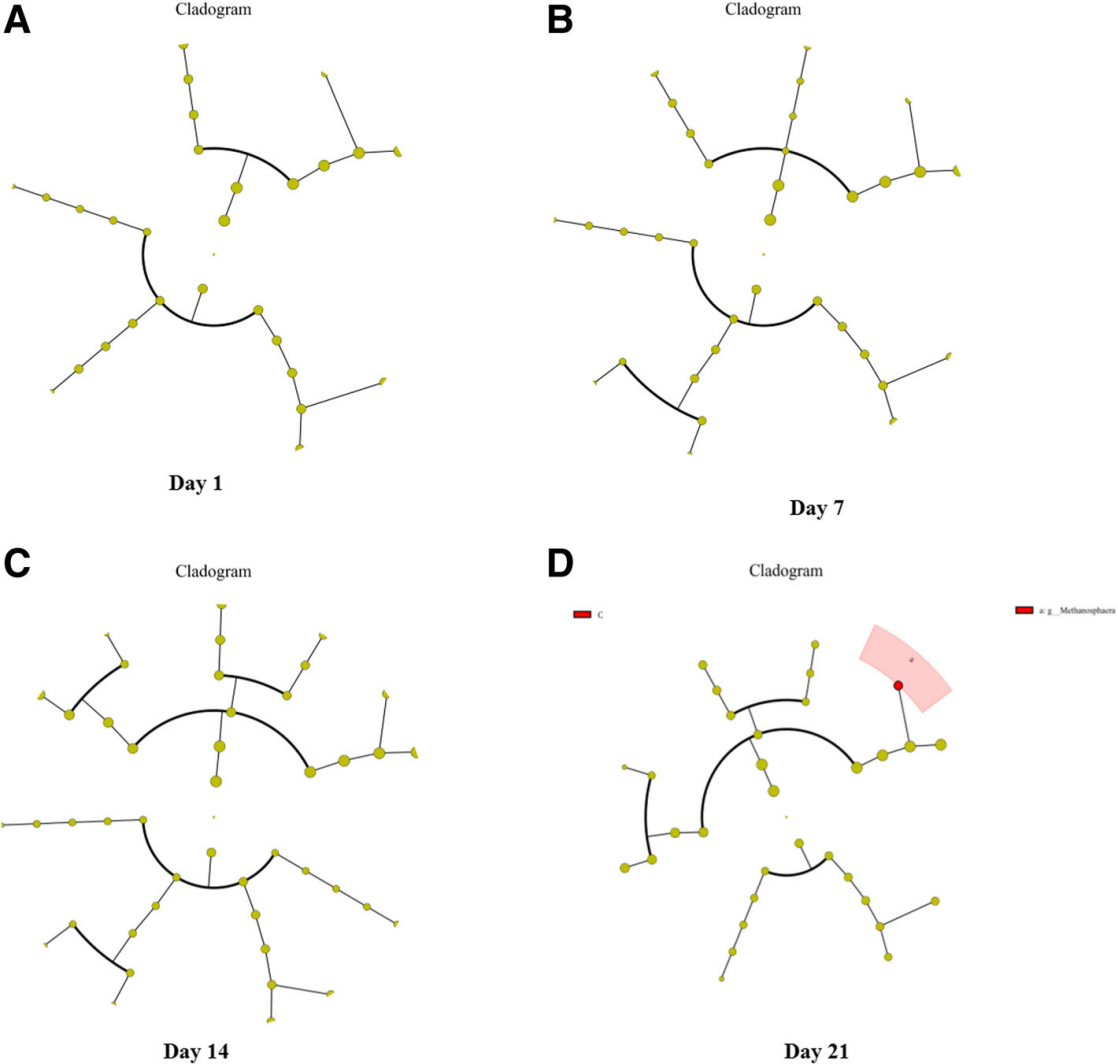
Cladograms, generated from LEfSe analysis, represent taxa enriched in Con (blue) or Dex (red) methanogens. The central point represents the root of the tree (methanogens), and each ring represents the next lower taxonomic level (phylum through genus). The diameter of each circle represents the relative abundance of the taxon. When full identification was not possible, g_ or s_ alone was used for genus or species, respectively. **a** Day 1 in rumen. **b** Day 7 in rumen. **c** Day 14 in rumen. **d** Day 21 in rumen

Dex did not affect the structure of the methanogen community, as the PCoA plot spots did not separate at any of the time points examined (Fig. 6a–d). Class level methanogens were not affected by Dex (Additional file 6: Figure S6A–D). None of the methanogens was affected, except *Methanosphaera*, which decreased on the final day of the Dex treatment (Fig. 7d).

## Discussion

In the present study, ten male goats were randomly divided into two groups. Dex was used to make chronic stress model in the study. The object is to investigate the influence of chronic stress on the diversity of mircrobiota in the goats. However, the results showed that the microbita in the rumen, ceacum, and colon can resistant stress, even it had a great impact on the physiological metabolism.

WBCs increased significantly in response to Dex treatment in the present study, which may be a marker of animal welfare, consistent with a previous study [23]. As shown previously [24, 25], chronic Dex treatment significantly decreased DMI and body mass of growing goats, which may be attributable to the high level of blood glucose. As food intake decreased, resources for fermentation lessened, pH of the ruminal fluid increased after Dex administration, especially 2 h after the morning feeding on days 1 and 14 of the Dex treatment. However, the concentrations of rumen VFAs, which are produced by ruminal microorganism as an energy supplement, were not affected by Dex at any time during the experiment. Ruminal VFAs were changed significantly after the morning feeding on day 21 of the Dex treatment caused by a decrease in the DMI, which may have also reduced body mass. pH, which is mainly influenced by lactic acid, did not decrease with the VFAs. However, the acetate/propionate ratio, which indicates different fermentation types, was unaffected [26]. None of the VFAs in the caecum or colon changed, as well as the acetate/propionate ratio, but colonic isovalerate concentration increased significantly and fecal butyrate decreased, leading to a significant decrease in total VFAs on days 14 and 21 of treatment. However, the acetate/propionate ratio remained unchanged. Ruminal urea and ammonia, as very important protein supplements, were not affected by Dex administration, suggesting that changes in body mass were not caused by the protein supplement. Increased plasma glucose and decreased plasma cortisol levels demonstrated that Dex stimulates gluconeogenesis and suppresses the cortisol secretion, consistent with previous studies [1, 27]. Taken together, our results reveal that Dex changed the goat’s metabolism.

Many studies have been performed about the interaction between the host and microbiota, and indicated that changes in the host’s metabolism affect the composition of microbiota [19, 22]. Stress can affect the composition of microbiota in rats [15, 28]. We used next-generation sequence technology to detect the composition and community structure of the microbiota.

In the present study, the Dex effect on microbial community composition was measured by unweighted UniFrac distances (β-diversity). The positions of the Dex and Con samples did not separate on the PCoA plot, suggesting that Dex did not affect the microbiota community structure. However, this result contrasts with a previous study in rats, showing that stress disrupted the microbiota community [15, 28]. This difference may have been caused by the organs sampled or species differences. Interestingly, the relative abundance of bacteroidetes dominated other bacteria on day 21 in the Dex group, whereas the Con group was dominated by firmicutes. Several changes in the microbial population were seen from the phylum to the genus levels. *Prevotella* promotes increased hepatic glycogen storage in mice [29], which was consistent with the present study (data not shown), and was the only bacteria that changed at each sampling time. The strictly anaerobic bacteria *Christensenella* induces inflammation [30, 31], and *Desulfovibrio*, which removes zinc from wastewater [32], increased in the Dex group after 7 days of treatment, indicating that inflammation and zinc metabolism were disrupted. *Prevotella* and *Selenomonas* are representative nitrate- and nitrite-reducing bacteria, respectively that increased in the Con group. Increased abundances of *Prevotella* and *Howardella*, are caused by the high-concentrate diet and a reduction of DMI [33], and *Ruminococcus*, a mucosa-associated bacteria linked with gastrointestinal disease [34], increased on day 14 in the Con group, suggesting inflammation in the Con group and the anti-inflammatory activity of Dex. *RC9_gut_group* and *Candidatus*, obligate anaerobic bacteria [35], and *Desulfobulbaceae* increased in the Con group on the last day of the experiment. *Prevotella* and *Clostridiaceae*, which play a role in pathogenesis [36], and *Treponema*, which shares a common ancestor with human pathogenic treponemes [37], increased in the Dex group on the last day of the experiment, demonstrating inflammation in the Dex group. Focusing on intestinal microbiota, the BS11_gut_group, family Bacteroidetes, was the only bacteria that increased in the caecum and colon after treatment, which is altered by a high-concentrate diet and reduced DMI [38]. *Parasutterella* increased in the caecum of the Con group. *Anaerostipes*, which utilize lactate [39] and *Butyrivibrio*, which ferment hemicelluloses [40], increased in the colon after treatment, demonstrated that fermentation was more effective in the Dex than in the Con groups. However, the VFAs did not change. *Anaeroplasma*, which is an obligate anaerobic bacterium [41], decreased in the colon after Dex treatment. Compared with a previous study [42], Dex increases total aerobes, total anaerobes, and *Lactobacilli*, but decreases *Coliforms*. However, we found contrasting results, which may have been caused by species differences.

These results suggest that Dex did not significantly affect the composition of the goat microbiota and only affected a few microbes. Those bacteria that were affected did not influence the fermentation type. We examined the changes in methanogenes, which produce methane and contribute to global warming [20]. The Dex and Con groups did not separate on the PCoA plot, suggesting that Dex did not disrupt the methanogen community structure, except *Methanosphaera*, which contains two species [43]. These results suggest that Dex did not affect the composition of methanogenes.

## Conclusions

Taken together, Dex affected metabolism, such as food intake and glycometabolism, in goats. However, it did not disrupt the microorganismal community structure in the rumen, caecum, or colon. The complexity of the rumen may be the reason for the differences in our results with previous studies, where microorganisms can resist the impact of the host, but have dramatic effects on metabolism.

## Methods

### Animals and experimental procedures

Ten healthy male goats (body weight 25 ± 1.0 kg) fitted with ruminal cannulas were raised in individual pens with free access to water and fed twice daily at 08:00 h and 18:00 h, respectively. Goats were offered free access to the same diet containing 43% corn, 5% wheat bran, 17% mixed concentrate and 35% forage. Animals were accustomed to all procedures of sampling and treatment before treatments for two weeks. The dose of Dex was determined based on the previous study by Emikpe et al. [44]. Ten goats were randomly assigned to two groups: one group was injected intramuscularly with Dex, as Group I (Dex-Dexamethasone; *n* = 5), 0.2 mg/kg, another group injected intramuscularly with the same volume saline (0.9%) as Group II (Con-Control; n = 5), injection at 7:30 before morning feeding for 21 days.

### DMI measurement, plasma collection, and body weight

Dry matter intake (DMI) was detected every day, before morning feeding. At 1, 7, 14, and 21 day, blood was sampled using heparin-containing vacuum tubes from jugular vein. Then 4 °C and 800×g for 10 min centrifugation, extracted plasma in eppendorf and stored at − 20 °C. At 14 and 21 day more blood samples were taken by using ethylenediaminetetraacetic acid containing vacuum tubes from jugular for blood routine examination. At the 21 day, the body weight had been measured.

### Feces, ruminal fluid, colonic and caecal content collection and assay

We combined and adapted previously described methods to analyze feces, ruminal fluid, colonic and caecal content collection [26, 45, 46]. Feces were collected after morning feeding at 1, 7, 14, and 21 day, shortly after collection, 5 g of fresh feces was dissolved with 5 mL of water. A portion of the extract was centrifuged at 2000×g for 10 min, and collected the supernatant. Then 1 mL of freshly prepared 25% metaphosphoric acid was added to 4 mL of the supernatant. The samples were then centrifuged (17,000×g for 10 min), and the supernatant fluid was stored at − 20 °C prior to the determination of volatile fatty acids (VFAs).

Samples of ruminal contents (approx. 50 mL) were obtained on days 1, 7, 14, and 21 of the experiment and at 0, 2, 4, and 6 h after morning feeding. Contents of the rumen (2 mL) collected at 0 h during 4 days were stored at liquid nitrogen immediately, were used for DNA extraction. Other ruminal samples were strained through four layers of cheesecloth. The pH of the ruminal fluid was measured immediately after collection by a mobile pH meter. About 2 mL of freshly prepared 25% metaphosphoric acid was added to 8 mL of strained ruminal fluid. The samples were then centrifuged (17,000×g for 10 min), and the supernatant fluid was stored at − 20 °C prior to the determination of VFAs. The remaining ruminal fluid samples were centrifuged at 12000×g for 15 min at 4 °C, and stored at − 20 °C until analysis.

At the end of the experiment, goats were slaughtered after overnight fasting. All goats were weighed and killed with neck vein injections of xylazine [0.5 mg/kg body weight; Xylosol; Ogris Pharme, Wels, Austria] and pentobarbital [50 mg/kg body weight; Release; WDT, Garbsen, Germany]. After death, the hind gut mucosal tissues were carefully removed. The content of caecum and colon were collected (approx. 50 mL). 2 mL of the colonic and caecal content were stored at liquid nitrogen were used for DNA extraction. The remaining of colonic and caecal samples were centrifuged at 12000×g for 15 min at 4 °C, supernatant was collected. About 1 mL of freshly prepared 25% metaphosphoric acid was added to 4 mL of supernatant from caecum and colon. Then samples were centrifuged (17,000×g for 10 min), and the supernatant fluid was stored at − 20 °C prior to the determination of VFAs.

### Blood parameters detection

Plasma glucose was measured using an automatic biochemical analyzer (7020, HITACHI, Tokyo, Japan). Cortisol was detected by using RIA cortisol kit (Beijing North Institute of Biological Tec.), strictly following the manufacturer’s instructions.

### Urea and ammonia detection

The rumen fluid samples were taken out of − 20 °C refrigerator. Then defreeze samples in room temperature. Berthelot (phenol-hypochlorite) reaction was used to determine NH3-N concentration [47]. The discetyl monoxime method was used to detect Urea-N level by a commercial kit (Nanjing Jiancheng Co., Nanjing, China).

### DNA extraction and 16S rRNA gene amplicon pyrosequencing

The methods of DNA extraction and 16S rRNA gene amplicon pyrosequencing were described previously [26]. 2 mL fluid from each goat collected was used for DNA extraction. The DNA samples were stored at − 80 °C until further processing. DNA purity was verified through agar gel electrophoresis. And the DNA was used as template to amplify the 16S V3-V4 region, amplificated with specific primers with Barcode. The primers used for the bacterial 16S rRNA hypervariable region (V3–V4) were 343F (5′-TACGGRAGGCAGCAG-3′) and 798R (5′-AGGGTATCTAATCCT-3′) [48], and the primers for archaeal 16S rRNA hypervariable region (V4–V5) were Arch519F (5′-CAGCMGCCGCGGTAA-3′) and Arch915R (5′-GTGCTCCCCCGCCAATTCCT-3′) [49]. The efficient hi-fi PCR enzyme and the Phusion® High-Fidelity PCR Master Mix with GC Buffer (New England Biolabs) were added to insure the amplification efficiency and accuracy. Then we made the database with the TruSeq® DNA PCR-Free Sample preparation Kit, and then used HiSeq2500 PE250 to sequencing.

Raw tags were got by the FLASH (version 1.2.7). Further, high quality clean tags were obtained through strict filtering processing by the Quantitative insights into microbial ecology (Qiime) (version 1.7.0). Effective tags were clustered to the Operational Taxonomic Units (OTUs) by Uparse (version 7.0.1001). The RDP classifier (version 2.2) and GreenGene database were used to species annotation. The unweighted principal component analysis (PCoA) analysis was made by the R (version 2.15.3). Linear discriminant analysis effect size (LEfSe) was used to detect significant changes in relative abundance of microbial taxa (LDA > 2).

### Statistical analysis

Data are presented as means ± SE. The data were tested for normal distribution and analyzed by Student’s unpaired t test using SPSS software packages (SPSS version 19.0 for Windows; SPSS Inc., Chicago, IL, USA). Data were considered statistically significant when *p* < 0.05. The numbers of replicates used for statistics were noted in the figures.

## Additional files

Additional file 1: **Figure S1**. Dynamic alteration of VFAs in rumen fluid from goat treated with Dex or Con, at 1, 7, 14, 21 day, after morning feeding. *A*. Day 1. *B*. Day 7. *C*. Day 14. *D*. Day 21. Means ± SE are plotted; ^#^*p* < 0.10, *p < 0.05, **p < 0.01 versus Con group. (TIF 12562 kb)

Additional file 2: **Figure S2**. Dynamic alteration of VFAs in feces from goat treated with Dex or Con, at 1, 7, 14, 21 day, after morning feeding. *A*. Day 1. *B*. Day 7. *C*. Day 14. *D*. Day 21. Means ± SE are plotted; ^#^*p* < 0.10, *p < 0.05, **p < 0.01 versus Con group. (TIF 424 kb)

Additional file 3: **Figure S3**. Dynamic alteration of urea in rumen from goat treated with Dex or Con, at 1, 7, 14, 21 day, after morning feeding. A. Day 1. B. Day 7. C. Day 14. D. Day 21. Means ± SE are plotted; #*p* < 0.10, *p < 0.05, **p < 0.01 versus Con group. (TIF 10055 kb)

Additional file 4: **Figure S4**. Dynamic alteration of ammonia in rumen from goat treated with Dex or Con, at 1, 7, 14, 21 day, after morning feeding. A. Day 1. B. Day 7. C. Day 14. D. Day 21. Means ± SE are plotted; #*p* < 0.10, *p < 0.05, **p < 0.01 versus Con group. (TIF 9728 kb)

Additional file 5: **Figure S5**. Altering compositions of microbial phylum (as a percentage of the total sequence), in rumen, caecum, and colon from goat treated with Dex or Con. *A*. Day 1 in rumen. *B*. Day 7 in rumen. *C*. Day 14 in rumen. *D*. Day 21 in rumen. *E*. Content of caecum. *F*. Content of colon. (TIF 8329 kb)

Additional file 6: **Figure S6**. Altering compositions of methanogenic class (as a percentage of the total sequence), in rumen, caecum, and colon from goat treated with Dex or Con. *A*. Day 1 in rumen. *B*. Day 7 in rumen. *C*. Day 14 in rumen. *D*. Day 21 in rumen. *E*. Content of caecum. *F*. Content of colon. (TIF 13224 kb)

## Abbreviations

Dex: Dexamethasone
DMI: Dry matter intake
OTU: Operational taxonomic unit
PCoA: Principal component analysis
VFA: Volatile fatty acid
